# Bone and Joint Infection Involving *Corynebacterium* spp.: From Clinical Features to Pathophysiological Pathways

**DOI:** 10.3389/fmed.2020.539501

**Published:** 2021-01-21

**Authors:** Pierre Chauvelot, Tristan Ferry, Virginie Tafani, Alan Diot, Jason Tasse, Anne Conrad, Christian Chidiac, Evelyne Braun, Sébastien Lustig, Frédéric Laurent, Florent Valour

**Affiliations:** ^1^Departement of Infectious Diseases, Hospices Civils de Lyon, Lyon, France; ^2^French Regional Reference Center for Complex Bone and Joint Infection (CRIOAc), Hospices Civils de Lyon, Lyon, France; ^3^International Centre for Research in Infectiology, INSERM U1111, Claude Bernard Lyon 1 University, Lyon, France; ^4^BioFilm Control, Saint-Beauzire, France; ^5^Orthopedic Surgery Unit, Hospices Civils de Lyon, Lyon, France; ^6^Laboratory of bacteriology, French National Reference Centre for Staphylococci, Hospices Civils de Lyon, Lyon, France

**Keywords:** *Corynebacterium*, osteoblasts, biofilm, bone and joint infection, intracellular

## Abstract

**Introduction:** Corynebacteria represent often-neglected etiological agents of post-traumatic and/or post-operative bone and joint infection (BJI). We describe here clinical characteristics and bacteriological determinants of this condition.

**Methods:** A retrospective cohort study described characteristics, outcome and determinants of treatment failure of all patients with proven *Corynebacterium* spp. BJI (i.e., ≥2 culture-positive gold-standard samples). Available strains were further characterized regarding their antibiotic susceptibilies, abilities to form early (BioFilm Ring Test®) and mature (crystal violet staining method) biofilms and to invade osteoblasts (gentamicin protection assay).

**Results:** The 51 included BJI were mostly chronic (88.2%), orthopedic device-related (74.5%) and polymicrobial (78.4%). After a follow-up of 60.7 weeks (IQR, 30.1–115.1), 20 (39.2%) treatment failures were observed, including 4 *Corynebacterium*-documented relapses, mostly associated with non-optimal surgical management (OR 7.291; *p* = 0.039). Internalization rate within MG63 human osteoblasts was higher for strains isolated from delayed (>3 months) BJI (*p* < 0.001). Infection of murine osteoblasts deleted for the β1-integrin resulted in a drastic reduction in the internalization rate. No difference was observed regarding biofilm formation.

**Conclusions:** Surgical management plays a crucial role in outcome of BJI involving corynebacteria, as often chronic and device-associated infections. Sanctuarisation within osteoblasts, implicating the β1 cellular integrin, may represent a pivotal virulence factor associated with BJI chronicity.

## Introduction

Bone joint infection (BJI), and especially prosthetic joint infection (PJI), represents a major public health concern (1), due to: (i) their prevalence, complicating 1 to 2% of arthroplasty procedures, with an important upcoming increase due to the projected rise in prosthetic joint replacement indications in the coming years (2, 3); (ii) their severity, associated with a 5% mortality rate and responsible for permanent disabilities in up to 40% of patients; and iii) their substantial economic burden estimated to be as high as 75,000 to 100,000 USD per episode attributed to protracted hospital course, re-operations, lengthened rehabilitation time and extended use of antimicrobials (4–7). Consequently, BJI has been pointed out as a priority axis of clinical and scientific research in many countries. The optima management requires a multidisciplinary approach combining both surgical procedure and extended antimicrobial therapy (8). Despite this complex management, they are associated with a high failure rate, exceeding 20% in some series, with frequent relapses and transition to a chronic state (9–14). This propensity to chronicity and relapse has been related to specific bacterial phenotypes responsible for subsequent emergence of bacterial reservoirs, protecting the pathogen from the extracellular host defenses and most antimicrobials (15, 16). These mechanisms have been well-characterized among *Staphylococcus aureus*, the main etiological agent of BJI (17–19), and consist in: (i) biofilm formation, an surface-adherant bacterial community living in a matrix of self-generated polymeric substances (20, 21); (ii) internalization and persistence within non-phagocytic bone cells, triggered by the interaction of staphylococcal fibronectin binding proteins (FnBP) with host Fibronectin that acts as a bridge with cellular α5β1 integrin to prompted bacterial endocytosis by an active cellular process (22–24); and (iii) phenotype switching to small colony variants (SCVs), a slow-growing bacterial phenotype which can emerge during intracellular or biofilm-associated lifestyles, and conferring enhanced resistance to antimicrobials (25, 26).

Corynebacteria are a highly heterogeneous group of Gram positive rods containing more than 110 species. Their pathogenic potential is species-dependent: some of them, as *Corynebacterium glutamicum* or *Cladosporium halotolerans*, have never been described in human pathology, when others have been implicated in various infectious disease, from urinary tract infection to infective endocarditis (27). Two type of virulence factor have been well-characterized in this genus. First, exotoxin production has been described in *Corynebacterium diphteriae, Corynebacterium ulcerans*, and *Corynebacterium pseudotuberculosis*. These three pathogenic strains can product diphteria toxin and/or phospholipase B, and therefore cause diphtheria, which is the best known corynebacteria-associated disease (27). Interestingly, even non-toxinogenic strains of *C. diphteriae* can cause invasive infections such as endocarditis, brain abscess or BJI (28). Secondly, some species have been shown to produce various adhesion molecules allowing interaction with eukaryote cells. A fibrinogen and fibronectin binding-like activity has been demonstrated from invasive strains of *Corynebacterium pseudodiphtericum* (29), interaction with fibronectin determines corynebacteria adhesion to vaginal epithelial cells (30), and *C. diphteriae* can invade epithelial cells, with an important role of a transmembrane protein called DIP0733, which possesses a fibrinogen and collagen binding activity (31–33).

As part of normal human skin microbiota, corynebacteria can be implicated in inoculation disease. They are especially involved in up to 3% of BJI (34–36). However, little is known about the specific aspects of *Corynebacterium* spp. BJI: epidemiologic data are lacking, their specific management is not addressed in current guidelines, and the pathophysiology of *Corynebacterium* spp. BJI has not been investigated so far (18, 37, 38). We report here the experience of our regional reference center with the management of *Corynebacterium* spp. BJI, aiming to describe patients' characteristics and treatment failure's determinants. Clinical isolates were further characterized for species distribution, antimicrobial susceptibility profile, ability to form biofilm and to invade bone cells.

## Patients and Methods

### Ethical Statements

This study (ClinicalTrials.gov registration number NCT03081273) received the approval of the French South-East Ethics Committee (reference number QH20/2014). All patients received written information about the study. The requirement for written informed consent was waived by the Committee for the protection of persons (CPP) according to French legislation at time of the study.

### Inclusion Criteria and Data Collection

This retrospective cohort study (2007–2016) included all patients followed-up in the infectious disease department of our tertiary care center for a proven *Corynebacterium* spp. BJI, i.e., with clinical, biological and/or radiological symptoms consistent with the diagnosis of BJI, with at least two *per* operative culture-positive samples yielding the same isolate (same species and same antibiotic susceptibility profile), and treated as such (1, 37, 38). Patients with diabetic foot- or pressure ulcer-related osteomyelitis were excluded because of their specific pathophysiology and management. For each patient, data were extracted from medical records by two of the study authors (infectious diseases specialists).

Microbiological diagnosis was performed according to international standards. For each patient, three to five intraoperative bone and/or periprosthetic tissue samples were collected under sterile conditions. They were then inoculated onto a Columbia sheep's blood agar plate (with reading at days 1, 2, and 3 before being thrown away), two PolyVitex chocolate agar plates (with reading at days 1, 2, and 3 before being thrown away for the first one and with reading at days 7 and 10 for the second one), two blood agar plates for anaerobic incubation (with reading at days 3 and 5 before being thrown away for the first one and with reading at days 7 and 10 for the second one) and into a Schaedler anaerobic liquid broth for which a daily reading was performed. If not cloudy, the broth was systematically subcultured on day 10 onto chocolate and blood agar plates for anaerobic incubation, incubated for 5 days in 5% CO_2_ and anaerobic atmosphere, respectively. Isolated bacteria were identified according to standard laboratory procedures (VITEK 2 system or VITEK matrix-assisted laser desorption/ionization time-of-flight mass spectrometry; bioMerieux, Marcy l'Etoile, France). When several specimens were positive, the identification of each type of colony was performed for all specimens. Antimicrobial susceptibility profiles were determined at least twice for each type of bacteria after a random selection among the positive specimens. Results of superficial and/or soft tissue samples were excluded.

### Definitions

BJIs were classified according to: (i) the potential presence of an orthopedic implant (i.e., joint prosthesis or osteosynthesis device); and (ii) the duration of progression from the presumed date of inoculation (i.e., date of device implantation for post-operative ODI, or date of symptom onset for native BJI) up to diagnosis, differentiating acute ( ≤ 4 weeks) vs. chronic (>4 weeks), and early (≤ 3months) vs. delayed (>3 months) infections (1, 19).

The surgical strategies considered as optimal were: (i) surgical debridement for chronic osteomyelitis; (ii) debridement with implant retention for acute ODI; and (iii) implant removal for chronic ODI. One-time exchange for chronic ODI was accepted if bacterial identification was previously known, without compromised local conditions (sinus tract, abscess and/or flap coverage requirement) (3, 11).

Treatment failure consisted in: (i) clinically persisting infection under appropriate antibiotherapy; or (ii) clinical relapse after the end of antibiotherapy; or (iii) septic indication for unplanned surgical revision more than 5 days after primary procedure; or (iv) superinfection; or (v) death related to the BJI or to a complication of its management.

Biological inflammatory syndrome referred to a plasmatic CRP level > 10 mg/L.

### Strain Characterization and Susceptibility Testing

Baseline strain characterization was routinely performed at time of diagnosis and retrieved from patients' medical records, including (i) species identification using VITEK®2 MS (bioMérieux, version 2.8.4.20081127, Shimadzu Biotech) (39); and (ii) antimicrobial susceptibility profile using the disk diffusion method on Mueller-Hinton agar supplemented with 5% sheep blood, as recommended by the European Committee on Antimicrobial Susceptibility Testing. Most clinical isolates responsible for a BJI diagnosed at our institution had been stored in cryotubes at −80°C since 2007. Available *Corynebacterium* spp. strains isolated from the included patients were subcultivated on Colombia agar supplemented with 5% sheep blood (COS, bioMérieux, Marcy l'Etoile, France) at 37°C for 48 h for further bacteriological assessments.

### Biofilm Formation

Early-stage biofilm formation was assessed using a protocol based on the BioFilm Ring test®, relying on the immobilization of magnetic beads by the growing biofilm matrix (13). Briefly, 96-well microplates were inoculated with a set of 10-fold serial dilution of standardized bacterial suspension in BHI mixed with 1% (v/v) toner solution containing magnetic beads (Biofilm Control, Saint Beauzire, France). A well without bacteria was used as negative control. After 5 h of static incubation at 37°C, each well was covered with 100 μL of white opaque oil (contrast liquid) and plates were placed for 1 min on a dedicated block for magnetization before being scanned with a specific plate reader (Pack BIOFILM, Biofilm Control): free beads were attracted at the center of the well to form a spot, of which intensity dropped down as beads were immobilized during biofilm formation. The adhesion strength of each strain was expressed as BioFilm Index (BFI), as previously described (40). The biofilm-forming potential (BP) was calculated using the formula: BP = [1 – (BFI sample/average BFI of negative control)] for each well. The cut-off value corresponded to three standard deviations above the mean of the negative control wells (BFIc = 0.53). Isolates with values of BP above 0.53 were considered significant biofilm formers. The last dilution above 2BFIc identifies the ability of the microorganism to form biofilm: poor (BP < 2BFIc at 10^−1^ dilution), weak (BP > 2BFIc at 10^−1^ and/or 10^−2^ dilution), moderate (BP > 2BFIc at 10^−3^ and/or 10^−4^ dilution), and high (BP > 2BFIc at 10^−5^ and/or 10^−6^ dilution) biofilm producers (41).

Ability to form mature biofilm was evaluated using the crystal violet staining test, as previously described (42). Briefly, 96-well microplates were inoculated with standardized bacterial suspension in BHI supplemented with 1% glucose, and incubated for 24 h at 37°C. After being washed, biofilm was colored with 100 μL of 0.1% crystal violet (Merck, Fontenay-sous-Bois, France). After new wash, dye bound to the biofilm was resolubilized with 100 μL of 33% acetic acid (VWR International) per well. The optical density at 490 nm, measured with a micro ELISA Auto Reader, Model 680 (BioRad, Hercules, USA), allows a quantitative measurement of formed biofilm. *S. aureus* 6850 was used as positive control in each experiment.

### Invasion of Human Osteoblasts

The ability of gentamicin-susceptible isolates to invade osteoblasts was evaluated in a gentamicin-protection assay. MG63 osteoblastic cells (CRL-1427; LGC standard, USA) were seeded at 40,000 cells per well into 48-well tissue culture plates and cultured for 24 h. Osteoblasts were infected with bacterial suspensions standardized in BHI at a multiplicity of infection of 1:100. After 2 h of co-culture, cells were treated for 1 h with gentamicin (200 mg/L) to kill the remaining extracellular bacteria and subsequently lysed by a 10-min incubation in sterile water. Dilutions of cell lysates were spiral-plated on COS using an easySpiral® automated plater (Interscience, Saint-Nom-la-Bretèche, France). Colonies were enumerated using a Scan®1200 automated plate reader (Interscience).

Given that the internalization of *S. aureus* within osteoblasts requires bacterial binding to the cellular α5β1 integrin via fibronectin (43, 44), *Corynebacterium* internalization was further investigated by infecting two murine osteoblastic cell lines with isolates able to invade MG63 cells in the above model: (i) OB-β1^+/+^ expressing a functional integrin β1 subunit, (ii) OB-β1^−/−^ deficient in the expression of the β1 integrin subunit after the conditional deletion of the *itgb1* gene by transfection (45, 46).

*S. aureus* laboratory strain 6850 was used as positive control in each experiment while *S. aureus* DU5883 strain, deleted for the *fnbA/B* genes (and so unable to invade osteoblasts), was used as negative control (47).

### Statistical Analysis

Studied variables were described as percentages for dichotomous variables and as medians with interquartile range (IQR) for continuous variables. In percentage calculation, the number of missing values was excluded from the denominator. Non-parametric tests were used to compare groups (Fisher exact and Mann-Whitney U tests), as appropriate. Kaplan-Meier curves were compared between groups using the log-rank (Mantel-Cox) test. Determinants of treatment failure were assessed using stepwise binary logistic regression, and expressed as odd ratios (ORs) with their 95% confidence intervals (95%CI). Non-interacting variables with medical meaning and *p*-values obtained in univariate analysis < 0.15 were included in the final multivariate model. Bacteriological data provide from three independent experiments in triplicate, and results are expressed as mean of the nine measure points and its 95%CI. Results were expressed relatively to *S. aureus* 6850. A value of *p* < 0.05 was considered significant. All analyses were performed using SPSS v19.0 (SPSS, Chicago, IL, USA) and GraphPad-Prism v5.03 (GraphPad, San Diego, CA, USA) softwares.

## Results

### Characteristics of the Included Population

Fifty-one *Corynebacterium* spp. BJIs occurring in 49 patients were included, as two patients presented two consecutives independent BJI episodes (Table 1). All infections resulted from an inoculation mechanism, and were mostly chronic (*n* = 45, 88.2%) and ODI (*n* = 38, 74.5%). ODI included 23 (60.5%) osteosynthesis devices and 15 (39.5%) prosthetic joint infections (PJI) (Table 2).

**Table 1:** Comparison of patients with favorable and unfavorable outcome and determinants of treatment failure in all patients with *Corynebacterium* spp. BJI (univariate analysis).

	**Outcome**				**Univariate analysis**	
	**Total population**	**Favorable**	**Failure**	***p*-value**	**OR (95%CI)**	***p*-value**
***n***	**51**	**31**	**20**			
**Demographics**						
Male gender	36 (70.6%)	19 (61.3%)	17 (85.0%)	0.115	3.579 (0.861–14.871)	0.079
Age (median, 95%CI), years	54.2 (44.2–68.8)	52.5 (46.3–67.6)	56 (41.6–69.0)	0.862	0.996 (0.963–1.030)^*^	0.810
**Comorbidities**						
BMI (median, 95%CI), kg/m^2^	26.9 (23.5–28.6)	25.3 (23.7–27.5)	27.3 (23.3–28.8)	0.378	1.112 (0.938–1.319)	0.220
ASA score (median, 95%CI)	1 (1–2)	2 (1–2)	1 (1–2)	0.697	0.908 (0.477–1.729)	0.770
CCI (median, 95%CI)	0 (0–2)	0 (0–2)	0.5 (0–2)	0.687	1.068 (0.737–1.547)	0.728
***Corynebacterium*** **species**						
*C. striatum*	18 (37.5%)	12 (38.7%)	6 (36%)	0.764	0.7731 (0.218–2.444)	0.611
*C. tuberculostearicum*	6 (12.5%)	3 (9.7%)	3 (17.6%)	0.661	1.750 (0.315–9.716)	0.522
*C. simulans*	5 (10.4%)	4 (12.9%)	1 (5.9%)	0.637	0.375 (0.039–3.633)	0.397
*C. jekeium*	4 (8.3%)	2 (6.5%)	2 (11.8%)	0.629	1.706 (0.220–13.243)	0.610
*C. minutissimum*	4 (8.3%)	2 (6.5%)	2 (11.8%)	0.629	1.706 (0.220–13.243)	0.610
*C. amycolatum*	3 (6.3%)	3 (9.7%)	0 (0.0%)	1.000	0.519 (0.050–5.379)	0.582
*Corynebacterium urealyticum*	2 (4.2%)	1 (3.2%)	1 (5.9%)	1.000	1.667 (0.098–28.320)	0.724
Others	5 (10.4%)	3 (9.7%)	2 (11.8%)	0.661	1.750 (0.315–9.716)	0.522
**Type of BJI**						
Native chronic osteomyelitis	13 (25.5%)	9 (29.0%)	4 (20.0%)	0.529	0.611 (0.160–2.339)	0.472
ODI						
PJI	15 (39.5%)	6 (27.3%)	9 (56.3%)	0.099	3.429 (0.078–13.390)	0.076
Osteosynthsesis device	23 (60.5%)	16 (72.2%)	7 (48.3%)	0.099	0.292 (0.075–1.139)	0.076
**BJI mechanism**						
Superinfection	24 (47.1%)	13 (41.9%)	11 (55.0%)	0.402	1.692 (0.545–5.257)	0.363
Inoculation mechanism	51 (100%)	31 (100%)	20 (100%)	1.000	NC	NC
Post-operative	48 (94.1%)	30 (96.8%)	18 (90.0%)	1.000	0.300 (0.025–3.549)	0.340
Post-traumatic	23 (45.1%)	14 (45.2%)	9 (45.0%)	0.553	0.994 (0.321–3.075)	0.991
**BJI chronology**						
Early infection (< 3 months)	34 (69.4%)	21 (70.0%)	13 (68.4%)	1.000	0.929 (0.268–3.219)	0.907
Chronic infection (>4 weeks)	45 (88.2%)	26 (83.9%)	19 (95.0%)	0.384	3.654 (0.394–33.880)	0.254
**Diagnostic features**						
Sinus tract	29 (63.0%)	18 (60.0%)	11 (68.8%)	0.750	1.467 (0.406–5.301)	0.559
Abscess	9 (20.0%)	5 (17.2%)	4 (25.0%)	0.700	1.600 (0.362–7.073)	0.535
Biological inflammatory syndrome	30 (69.8%)	17 (58.6%)	13 (92.9%)	0.033	9.176 (1.054–79.892)	0.045
Initial plasmatic CRP level (mg/L)	37.3 (15.3–96.2)	30.0 (14.5–91.5)	45.0 (21.7–93.9)	0.565	0.998 (0.990–1.007)	0.724
Polymicrobial infection	40 (78.4%)	26 (83.9%)	14 (70.0%)	0.304	0.449 (0.116–1.736)	0.246
**Surgical management**	47 (92.2%)	29 (93.5%)	18 (90.0%)	0.640	0.621 (0.080–4.804)	0.648
Inappropriate surgical management	12 (23.5%)	5 (16.1%)	8 (35.0%)	0.178	2.800 (0.743–10.553)	0.128
Flap coverage requirement	8 (15.7%)	3 (9.7%)	5 (25.0%)	0.237	3.111 (0.652–14.845)	0.155
**Medical management**						
Antimicrobial therapy duration						
Total treatment duration (weeks)	24.7 (14.1–54.4)	18.1 (13.1–33.9)	37.1 (22.4–59.4)	0.080	1.018 (0.997–1.039)	0.088
*Corynebacterium*-specific treatment duration	16.3 (13.1–22.8)	14.9 (12.9–18.9)	20.0 (16.0–33.9)	0.039	1.070 (1.004–1.141)	0.038
*Corynebacterium*-specific intravenous treatment	48 (94.1%)	29 (93.5%)	19 (95.0%)	1.000	0.310 (0.111–15.479)	0.830
Intravenous treatment duration	14.1 (6.5–18.3)	13.1 (5.9–15.0)	18.1 (14.9–27.9)	0.130	1.095 (1.007–1.190)	0.034
Oral switch	26 (54.2%)	20 (69.0%)	6 (31.6%)	0.018	0.208 (0.060–0.723)	0.013
*Corynebacterium*-specific combination therapy	36 (75.0%)	24 (82.8%)	12 (63.2%)	0.176	0.357 (0.093–1.365)	0.132
Combination therapy duration	12.9 (6.8–16.6)	12.1 (4.3–13.9)	19.3 (10.6–22.5)	0.491	1.107 (0.977–1.255)	0.112
**First line antimicrobial regimen**						
Initial oral antimicrobial therapy	18 (35.3%)	14 (45.2%)	4 (20.0%)	0.080	0.304 (0.082–1.119)	0.073
Betalactam	24 (50.0%)	15 (50%)	9 (50.0%)	1.000	1.000 (0.311–3.218)	1.000
Glycopeptide	35 (68.6%)	23 (74.2%)	12 (60.0%)	0.360	0.522 (0.157–1.738)	0.289
Clindamycin	5 (10.0%)	4 (13.3%)	1 (5.0%)	0.636	0.342 (0.035–3.311)	0.354
Linezolid	0 (0.0%)	0 (0.0%)	0 (0%)	NC	NC	NC
Daptomycin	3 (5.9%)	0 (0.0%)	3 (15.0%)	0.055	NC	NC
**Posterior antimicrobial regimen**						
Betalactam	20 (40.8%)	11 (36.7%)	9 (47.4%)	0.555	1.555 (0.484–4.995)	0.459
Glycopeptide	19 (37.3%)	10 (32.2%)	9 (47.4%)	0.358	1.718 (0.539–5.475)	0.360
Clindamycin	8 (15.7%)	5 (16.1%)	3 (15.0%)	0.496	0.555 (0.125–2.469)	0.439
Linezolid	9 (17.6%)	7 (22.6%)	2 (10.0%)	0.512	1.643 (0.442–6.102)	0.458
Daptomycin	5 (9.8%)	4 (12.9%)	1 (5.0%)	1.000	0.722 (0.119–4.372)	0.723
Daptomycin-containing regimen	8 (15.7%)	4 (12.9%)	3 (15.0%)	0.696	1.687 (0.370–7.697)	0.499

*95%CI, 95% confidence interval; ASA, American society of anesthesiologists; BMI, Body mass index; CCI, Charlson comorbidity index; CRP, C-reactive protein;NC, Not calculable; ODI, Orthopedic device-related infection; OR, Odd ratio; PJI, Prosthetic joint infection*.

* *Calculated for 10 additional years*.

**Table 2:** Comparison of patients with favorable and unfavorable outcome and determinants of treatment failure in patients with *Corynebacterium* spp. orthopedic device-related infection (univariate analysis).

	**Outcome**			**Univariate analysis**		
	**ODI**	**Favorable**	**Failure**	***p*-value**	**OR (IC95%)**	***p*-value**
**Demographics**						
Male gender	25 (65.8%)	12 (54.5%)	13 (81.3%)	0.165	3.611 (0.798; 16.347)	0.096
Age (median, 95%CI), years	53.1 (44.1;69.0)	52.2 (47.5;69.0)	54.0 (41.6;69.0)	0.679	0.991 (0.956; 1.027)	0.615
**Comorbidities**						
BMI (median, 95%CI), kg/m^2^	25.4 (22.9;28.6)	24.5 (22.5;26.0)	28.0 (23.5;28.9)	0.100	1.199 (0.969; 1.484)	0.095
ASA score (median, 95%CI)	1 (1.0;2.0)	1 (1.0;2.0)	1.5 (1.0; 2.3)	0.589	1.250 (0.605; 2.584)	0.547
CCI (median, 95%CI)	0 (0.0;1.8)	0 (0.0;1.0)	0.5 (0.0;2.0)	0.453	1.325 (0.809; 2.171)	0.263
**BJI mechanism**						
Superinfection	16 (42.1%)	8 (36.4%)	8 (50.0%)	0.511	1.750 (0.472; 6.483)	0.402
Inoculation mechanism	38 (100%)	22 (100%)	16 (100%)			
Post-operative	37 (97.4%)	22 (100%)	15 (93.8%)	0.421	NC	NC
Post-traumatic	16 (42.1%)	10 (45.5%)	6 (37.5%)	0.744	0.720 (0.193; 2.681)	0.624
**BJI chronology**						
Early infection (< 3 months)	25 (69.4%)	13 (61.9%)	12 (80.0%)	0.295	2.462 (0.527; 11.500)	0.252
Chronic infection (>4 weeks)	33 (86.8%)	18 (81.8%)	15 (93.8%)	0.374	3.333 (0.336; 33.113)	0.304
**Diagnostic features**						
Sinus tract	21 (63.6%)	13 (61.9%)	8 (66.7%)	1.000	1.231 (0.278; 5.454)	0.785
Abscess	6 (18.8%)	2 (10.0%)	4 (33.3%)	0.165	4.500 (0.679; 29.808)	0.119
Biological inflammatory syndrome	26 (78.8%)	14 (70.0%)	12 (92.3%)	0.202	5.143 (0.540; 48.943)	0.154
Initial plasmatic CRP level (mg/L)	30.0 (15.0;93.9)	20.0 (14.0;46.0)	52.4 (20.1;96.2)	0.414	0.999 (0.991; 1.007)	0.824
**Surgical management**	36 (94.7%)	22 (100%)	14 (87.5%)	0.171	NC	NC
Inappropriate surgical strategy	9 (25.0%)	4 (18.2%)	5 (35.7%)	0.147	3.500 (0.808–15.163)	0.094
Surgical strategy
DAIR/debridement	14 (38.9%)	7 (31.8%)	7 (50.0%)	0.314	2.143 (0.539; 8.512)	0.279
One-stage exchange	1 (2.8%)	1 (4.5%)	0 (0.0%)	1.000	NC	NC
Two-stage exchange	11 (30.6%)	9 (40.9%)	2 (14.3%)	0.142	0.241 (0.043; 1.346)	0.105
Definitive device ablation	10 (27.8%)	5 (22.7%)	5 (37.5%)	0.462	1.889 (0.430; 8.295)	0.400
Two-stage exchange OR definitive device ablation	21 (58.3%)	14 (63.6%)	7 (50.0%)	0.499	0.571 (0.147; 2.228)	0.420
Flap coverage requirement	5 (13.2%)	2 (9.1%)	3 (18.8%)	0.632	2.308 (0.338; 15.750)	0.393

*95%CI, 95% confidence interval; ASA, American society of anesthesiologists; BMI, Body mass index; CCI, Charlson comorbidity index; CRP, C-reactive protein; NC, Not calculable; ODI, Orthopedic device-related infection; OR, Odd ratio; PJI, Prosthetic joint infection*.

Surgery was performed in 47 (92.2%) patients and considered as optimal in 39 (76.5%) cases. The total duration of antibiotherapy specifically directed against corynebacteria was 18.1 (IQR, 13.1–29.3) weeks, initially administrated intravenously for 14.1 weeks (IQR, 6.5–18.3) in 48 patients (94.1%).

### Bacteriological Findings

**Figure 1:**
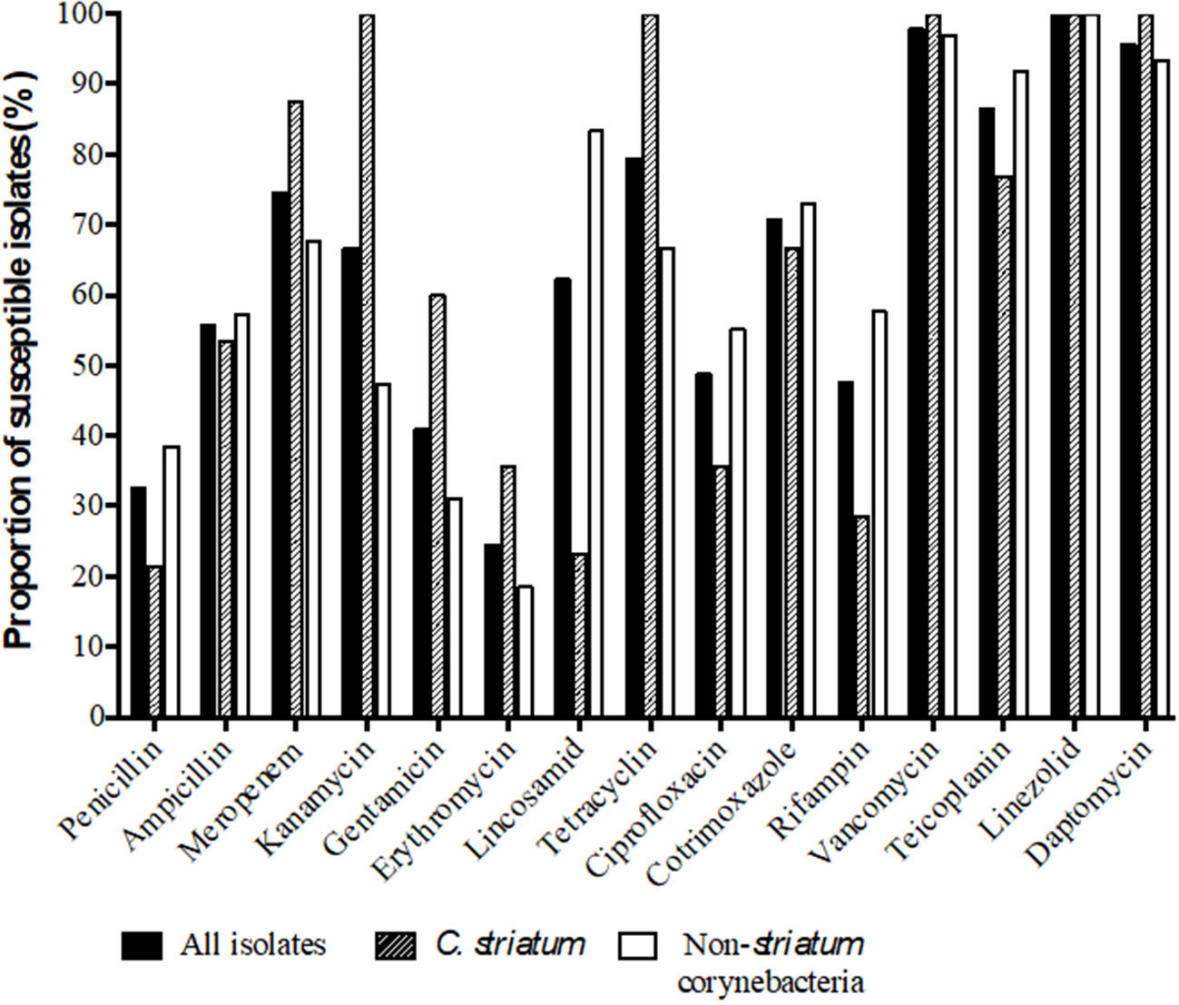
Susceptibility profile of the 51 BJI *Corynebacterium* spp. isolates.

As one patient presented a co-infection with two different *Corynebacteria*, 52 strains were considered for inclusion. Species identification and antimicrobial susceptibility testing were available in patients' medical records for 45 of them. The most frequent species were *Corynebacterium striatum* (*n* = 18, 37.5%) and *Corynebacterium tuberculostearicum* (*n* = 6, 12.5%). Antimicrobial susceptibility profiles are presented in Figure 1. Most infections were polymicrobial (*n* = 40, 78.4%), including co-infections with coagulase-negative staphylococci (*n* = 20, 50.0%), *Enterobacteriaceae* (*n* = 14, 35.0%), *S. aureus* (*n* = 8, 20.0%), anaerobes (*n* = 7, 17.5%), enterococci (*n* = 5, 12.5%), *P. aeruginosa* (*n* = 4, 10.0%), streptococci (*n* = 4, 10.0%) and/or *Candida* (*n* = 1, 2.5%). A detailed description of the eleven patients with a monomicrobial *Corynebacterium* spp. BJI is provided in Supplementary Table 1.

### Outcome and Determinants of Treatment Failure

**Figure 2:**
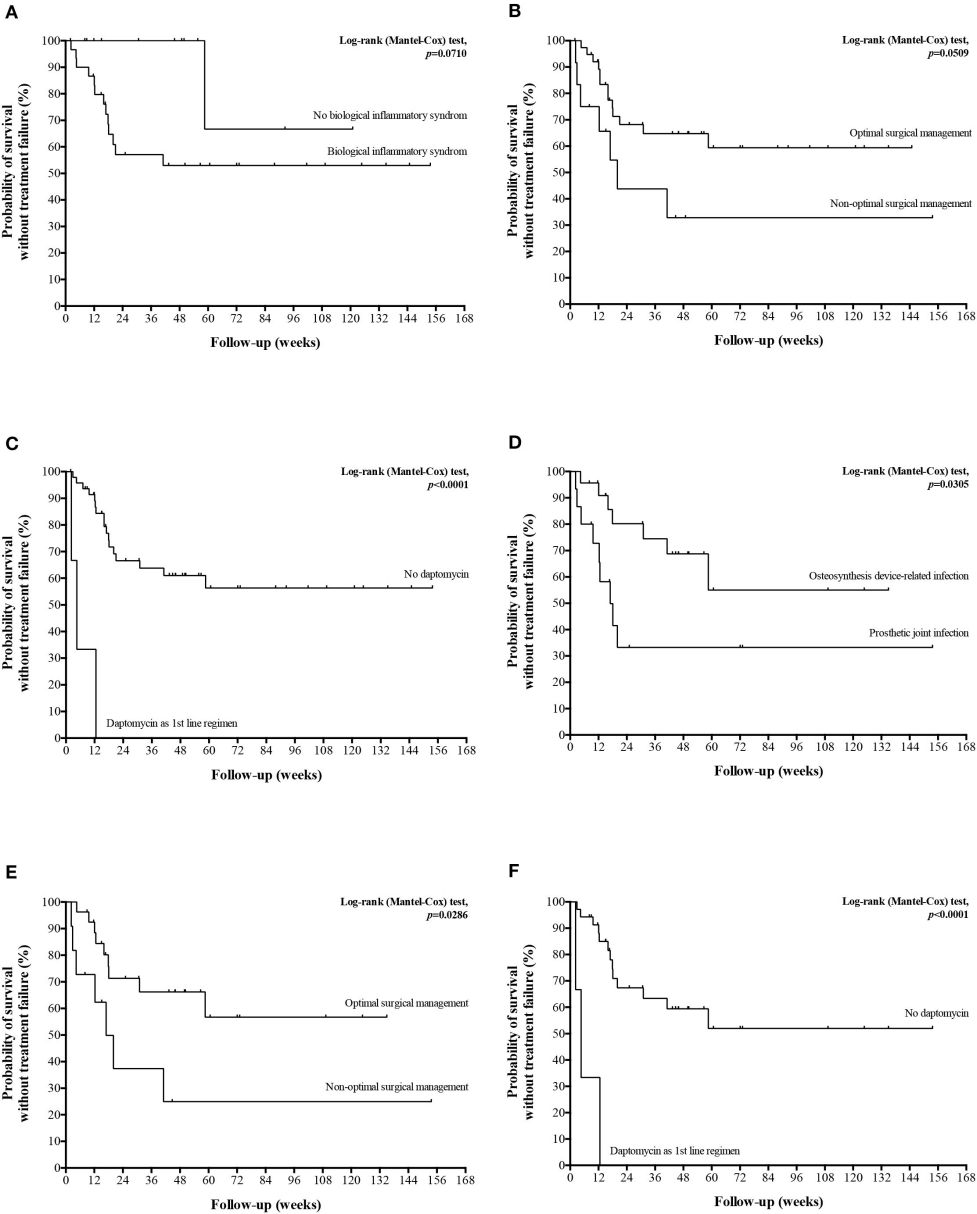
Kaplan-Meier curves for the cumulative risk of treatment failure in all patients **(A–C)** and in patients with ODI panel **(D–F)** according to the major determinants of treatment failure.

After a median follow-up of 60.7 weeks (IQR, 30.1–115.1) including 38.0 weeks (IQR, 10.1–85.7) after completion of the antibiotherapy, 20 (39.2%) treatment failures were observed in a median delay of 14.3 weeks (IQR, 9.1–18.6) after treatment initiation, including 13 (65.0%) persistent infections, 6 (30%) relapses, 10 (50%) superinfections and one infection-related death. Seventeen (85.0%) cases required an additional surgical procedure, including one limb amputation. Four (20.0%) treatment failures were documented with the same *Corynebacterium* spp. strain; no documentation was obtained in 8 (40.0%) patients. Comparison of patients with and without treatment failure is presented in Table 1, as well as univariate analysis for risk factor for treatment failure. In multivariate analysis, among male gender, initial biological inflammatory syndrome, non-optimal surgical management, and corynebacteria-directed combination therapy, independent determinants for treatment failure were an initial biological inflammatory syndrome (OR, 15.119; 95%CI, 1.189–192.205; *p* = 0.036) and non-optimal surgical management (OR, 7.291; 95%CI, 1.107–48.016; *p* = 0.039) (Figures 2A,B). Interestingly, the 3 (5.9%) patients who received daptomycin (6 to 8 mg/kg/day) as first-line regimen relapsed (Figure 2C), despite an optimal surgical management. Of note, two of these patients had a polymicrobial infection. The three *Corynebacterium spp*. Isolates were fully susceptible do daptomycin, with MICs of 0.5, 0.094, and 0.032 mg/L. The choice of daptomycin was based on the polymicrobial nature of the infection in one patient, and previous antimicrobial intolerances in the two others. Finally, daptomycin was used as part of a combination therapy in two of these three patients.

Concerning specifically ODI, 16 (42.1%) treatment failures were observed. No significant risk factors was highlighted (Table 2), but treatment failure-free survival curve analysis suggested a significantly poorer outcome in patients with PJI compared to osteosynthesis device infection, in case of non-optimal surgical management, and if daptomycin was used as first-line regimen (Figures 2D–F).

### Bone Cell Invasion

Among the 52 potential strains isolated from the patient study, 22 had not been conserved, five had been isolated in other institutions before patient referral to our reference center, seven were resistant to gentamicin preventing to perform gentamicin-protection assay, three could not be formally identified at the species level, and two *C. tuberculostearicum* strains had cultural aspect with tiny colonies preventing their enumeration on blood agar plates. Consequently, ability to invade human osteoblasts could be assessed for 13 corynebacteria strains (seven *C. striatum*, three *Corynebacterium simulans*, two *Corynebacterium amycolatum/xerosis*, and one *Corynebacterium minutissimum*) isolated from different patients (Table 3).

**Table 3:** Description of the isolates evaluated in the osteoblastic cell infection model and biofilm formation assays.

**Strain identification**	***Corynebacterium* species**	**Type of BJI**	**Chronology of infection**	Internalization rate (95%CI)^*^	**Biofilm-forming potential**	Mature biofilm formation (95%CI)^*^
Cor 1b^#^	*C. striatum*	Osteosynthesis infection	Early	0.34% (0.06–0.62)	POOR	0.36% (−0.08–0.79)
Cor 4	*C. striatum*	Native osteomyelitis	Early	3.63% (1.47–5.80)	POOR	35.71% (26.26–45.17)
Cor 5^#^	*C. simulans*	Prosthetic joint infection	Early	0.60% (0.30–0.89)	WEAK	1.64% (−0.84–4.13)
Cor 8b	*C. striatum*	Osteosynthesis infection	Delayed	4.29% (0.71–7.88)	WEAK	42.39% (30.65–54.13)
Cor 9b	*C. striatum*	Prosthetic joint infection	Early	1.30% (0.36–2.25)	POOR	5.66% (1.79–9.52)
Cor 10	*C. amycolatum/xerosis*	Prosthetic joint infection	Delayed	1.35% (0.37–2.32)	N/A	8.58% (3.78–13.38)
Cor 11a	*C. minutissimum*	Native osteomyelitis	Delayed	55.6% (28.99–82.24)	POOR	1.04% (−0.27–2.34)
Cor 12^#^	*C. simulans*	Prosthetic joint infection	Delayed	5.99% (3.11–8.87)	POOR	4.61% (−1.70–10.92)
Cor 13	*C. striatum*	Native osteomyelitis	Early	15.3% (9.67–20.91)	N/A	13.59% (6.11–21.06)
Cor 14	*C. simulans*	Native osteomyelitis	Delayed	2.84% (0.75–4.93)	POOR	8.56% (−0.04–17.15)
Cor 15	*C. amycolatum/xerosis*	Osteosynthesis infection	Delayed	206% (131.08–281.86)	POOR	18.06% (2.89–33.22)
Cor 16	*C. striatum*	Osteosynthesis infection	Early	35.2% (-3.23–73.647)	POOR	2.38% (−0.17–4.93)
Cor 18	*C. striatum*	Prosthetic joint infection	Delayed	7.29% (3.03–11.54)	POOR	0.01% (−0.01–0.04)

*95%CI, 95% confidence interval; BJI, Bone and joint infection; CO, Chronic osteomyelitis; ODI, Osteosynthesis device-associated infection; PJI, Prosthetic joint infection*.

* *Results are given as mean and its 95% confidence interval (95%CI), compared to S. aureus 6850*.

# *Designated monomicrobial infections*.

**Figure 3:**
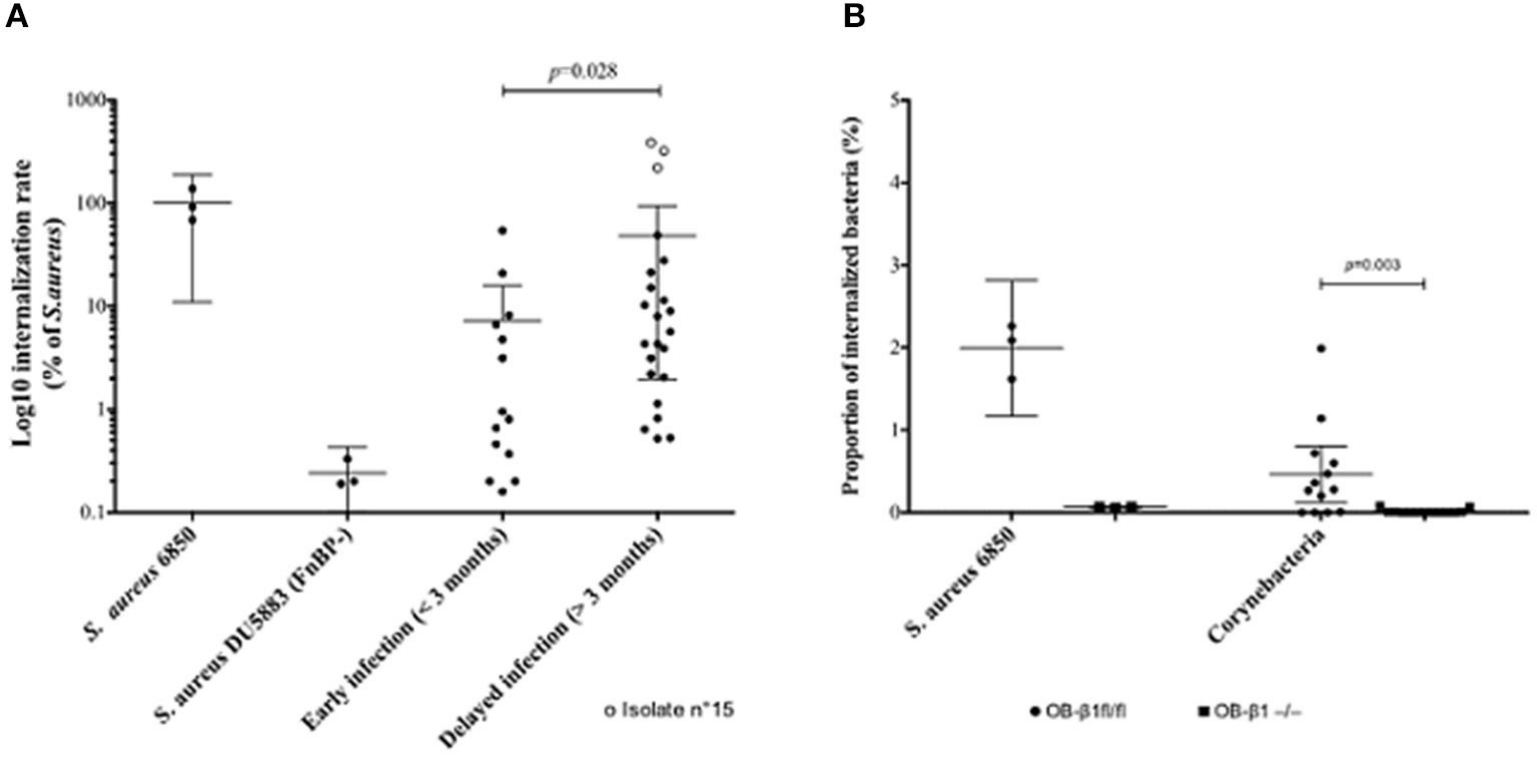
Ability of *Corynebacterium* spp. isolates to invade osteoblastic cells. **(A)** Internalization rates of *Corynebacterium* isolates in MG63 human osteoblasts according to bone and joint infection (BJI) evolution delay, in comparison with *S. aureus* 6850 (positive control) and *S. aureus* DU5883 strain, inactivated for the *fnbA/B* genes (FnBP, negative control). **(B)** Internalization rates of the *Corynebacterium* isolates in murine osteoblasts with functional (OB-β1^fl/fl^) or deficient (OB-β1^−/−^) expression of the integrin β1 subunit, in comparison with *S. aureus* 6850.

In comparison with *S. aureus* DU5883, all but one strain were significantly able to invade MG63 osteoblasts (Figure 3). The internalization rate was roughly comprised between 1 and 10% of positive control (*S. aureus* 6850). One *C. amycolatum/xerosis* strain (n°15) isolated from a delayed BJI even presented a very high internalization rate (200% of positive control).

Strains isolated from delayed BJI had a significantly higher internalization rate compared to early ones (Figure 3A).

The internalization rate of each species are provided in Supplementary Figure 1A. The little number of isolates per species did not allow to provide pertinent statistical comparison.

Strains able to invade MG63 human osteoblasts, including isolate n°15, were challenged in OB-β1^−/−^ murine osteoblasts, resulting in a drastic reduction of the internalization rate compared to OB-β1^+/+^ cells (Figure 3B).

### Biofilm Formation

Early-stage biofilm formation was assessed for 11 of the 13 isolates used in the cellular infection model. The two other Cor 10 and 13° formed aggregates under the culture conditions specifically required for the BioFilm Ring test®. All but two corynebacteria had a poor BP. The two last strains Cor 5 and Cor 8b had a weak BP (Table 3).

All the 13 isolates were evaluable regarding their mature biofilm formation by the crystal violet staining method. Six of them formed mature biofilm, with a rate ranging from 8.6 to 42.4% compared to *S. aureus* 6850 (Table 3).

The little number of isolates per species prevented providing relevant interspecies comparison (Supplementary Figure 1B).

Early or mature biofilm formation abilities were not correlated with any relevant clinical feature.

Of note, neither internalization nor biofilm formation ability had a significant impact on patient outcome.

## Discussion

Representing more than 3% of PJI etiologic agents (34–36), *Corynebacterium* spp. have been largely neglected in this field. Despite the limitations inherent to the retrospective and unicentric nature of our study, it provides major clinical and therapeutic insights regarding corynebacteria BJI. Our results are reinforced by the attempt to minimize the risk of considering commensal *Corynebacterium* spp. strains isolated as contaminants by including only BJI with at least two concordant positive *per* operative samples and excluding contiguous infections such as decubitus ulcer- and diabetic foot-related osteomyelitis that are associated with a high risk of sample contamination. Indeed, conclusions of some previously published series must be interpreted with caution as including more than 50% of patients with contiguous BJI and based on the culture results of superficial samples (48, 49).

Our results confirm that proven *Corynebacterium* BJI occur mainly by inoculation after trauma, mostly after road crash-related open fractures. Indeed, a predominance of young men was noted, with up to 70% of chronic osteomyelitis. PJI were less frequent than previously described (48), and mostly corresponded to superinfections during complex PJI managements. These differences can be explained by our stringent bacteriological definition of cases. Finally, the species distribution slightly differed from previous studies, with a predominance of *C. striatum* and *C. tuberculostearicum*, and less *C. amycolatum* and *Corynebacterium jekeium* than previously described (36, 48).

The management of BJI involving *Corynebacterium* is complex. First, optimal surgical management appeared as a crucial determinant for treatment outcome, as previously described for chronic and/or ODI (50, 51), including removal of orthopedic device and extensive bone curettage when necessary (37, 38). However, this theoretical optimal management can be impaired by fracture stabilization requirements, and sometimes leads to major tissue loss. Choice of antimicrobial therapy is also challenging. As shown by our results and previous series (48), *Corynebacterium* isolates can be resistant to most of the antibiotics commonly used in BJI, including amoxicillin which remains the drug of choice for susceptible isolates. Moreover, polymicrobial infection prevalence, vancomycin toxicity and/or patient's antibiotic intolerances can raise the need of off-label use of alternative drugs. In this setting, daptomycin has been increasingly used in Gram positive BJI (52). Interestingly, all patients treated by daptomycin experienced treatment failure, leading to highlight the use of this antimicrobial as a significant risk factor for poor outcome. Although based on a limited number of patients, this finding is coherent with treatment failures and daptomycin resistance selection observed during other chronic conditions such as infective endocarditis (53–55) and raises the question of reconsidering the use of daptomycin as a first-line agent. Finally, the higher rate of treatment failure of ODI compared to PJI was not explained by statistically significant differences between patients or their management. However, ODI mostly occurred following the management of severe limb trauma, requiring flap coverage in more than 20% of cases. Even if not highlighted by our results, the complexity of orthopedic situations observed in such kind of patients might have led to this poor outcome.

Overall, and despite a complex surgical and medical management, BJI with *Corynebacterium* spp. are difficult-to-treat infections, as evidenced by (i) the failure rate approaching 40%, (ii) the frequent need of iterative surgical procedures including surgical flap reconstruction in 15% of patients, and (iii) the prolonged courses of antimicrobial therapies. If polymicrobism has been highlighted as a risk factor for treatment failure in some studies (56), this point is still controversial (57), and polymicrobial infections were not associated with a poorer outcome in our series. Associated with a dramatically increase of morbidity and medical/societal cost (58), BJI chronicity and relapse have consequently to be investigated, including underlying mechanisms leading to bacterial escape from the action of the host immune system and/or the antibiotics. The extensive evaluation of *Staphylococcus aureus* BJI pathomechanisms highlighted three main phenotypic bacterial factors associated with BJI chronicity (59): (i)internalization and persistence in non-professional phagocytic bone cells (osteoblasts), which had been confirmed to be clinically associated with BJI chronicity (15), (ii) biofilm formation (60), and (iii) emergence of small colony variants (25). We provide here the first assessment of these mechanisms toward a collection of clinical *Corynebacterium* isolates responsible for BJI. We demonstrated that almost all *Corynebacterium* isolates were able to invade osteoblasts and that their internalization rate was correlated with BJI chronicity, even if *in fine* the cure rate was not impacted. This ability to sanctuarize in bone cells emphasized the importance of surgical debridement in chronic BJI with *Corynebacterium* spp. and pleads for including the ability of antibiotics to eradicate the intracellular reservoir of corynebacteria in the choice of antimicrobial therapy strategies, as suggested for *S. aureus* (15, 61, 62). Interestingly, the infection of murine osteoblasts deficient in the expression of β1 integrin abolished the cellular invasion ability of the evaluated strains. This strongly suggest that corynebacteria osteoblastic invasion relies on mechanisms similar to *S. aureus*, of which fibronectin binding proteins A and B link to fibronectin of the bone matrix that acts as bridges between *S. aureus* and osteoblasts through the cellular α5β1 integrin (43, 44). The ligand of the cellular β1 integrin remains to be described in corynebacteria, as representing a future potential therapeutic target. Regarding biofilm formation, all investigated strains of our study were poor biofilm formers as most Gram positive bacteria except *S. aureu*s (40). A few studies have however suggested that biofilm formation could be a determinant of *Corynebacterium* spp. hospital acquired infections (63, 64). Unfortunately, we were not able to perform the biofilm and intracellular assays on the whole series, which might represent a bias. Indeed, the ability of corynebacteria to form biofilm seems strain-related, as shown by the differences observed toward a same species according to their sequence types (ST) (63, 65). However, no clinical differences were noted in our series between the patients for which the strain was available and the others (data not shown). Additionally, the comparison of isolates coming from mono and polymicrobial infection would have been interesting, but only three strains of our series were isolated from monomicrobial infection, making the comparison irrelevant.

The short time of follow-up (less than a year) of patients without treatment failure is not enough to affirm treatment success and represent another limitation to this study. However, even if relapses have been described several months/years after the end of therapy, this represents a rare event.

This series of proven *Corynebacterium* BJI allows to better understand this neglected disease. Most often presenting as a post-traumatic or post-surgical chronic infection, this difficult-to-treat condition requires a complex and collaborative medical-surgical management due to its poor prognosis which is mostly driven by the initial surgical debridement. Furthermore, if biofilm formation did not appear as a pivotal physiopathological mechanism of *Corynebacterium* in BJI, bone cells invasion via the cellular β1 integrin allows the formation of an intracellular reservoir that leads to chronic infection.

## Data Availability Statement

All datasets generated for this study are included in the article/Supplementary Material.

## Ethics Statement

The requirement for written informed consent from participants for the usage of clinical isolates in the study was waived by the Committee for the protection of persons (CPP) according to French legislation at time of the study.

## Author Contributions

PC collected the data, conducted most of the experiment, and wrote the manuscript under the supervision of FV. FV designed the study, analyzed the results and helped to perform the experiments. TF and FL helped to design the study. VT and AD reviewed the manuscript. JT provided the protocols for biofilm experiments. AC, CC, EB, and SL provided clinical data. All authors revised and edited the manuscript and read and approved the final manuscript.

## Conflict of Interest

Biofilm Control provided support in the form of salaries for JT but did not have any additional role in the study design, data collection and analysis, decision to publish, or preparation of the manuscript. The remaining authors declare that the research was conducted in the absence of any commercial or financial relationships that could be construed as a potential conflict of interest.

## Supplementary Material

Supplementary Figure 1 Comparison of *Corynebacterium* species regarding their ability to invade MG63 human osteoblasts **(A)** and to form mature biofilm **(B)**.

Supplementary Table 1 Clinical characteristics of the eleven patients with monomicrobial *Corynebacterium* spp. BJI.

Supplementary Table 2 Description of patients according to the BJI types, and comparison of osteosynthesis device-related and prosthetic joint infection.
