# Supplementary figures and images for: Bone and Joint Infection Involving Corynebacterium spp.: From Clinical Features to Pathophysiological Pathways
Source: Front Med (Lausanne). 2021 Jan 21;7:539501. doi: 10.3389/fmed.2020.539501 (PMC7873945; doi:10.3389/fmed.2020.539501)

## Slide 1
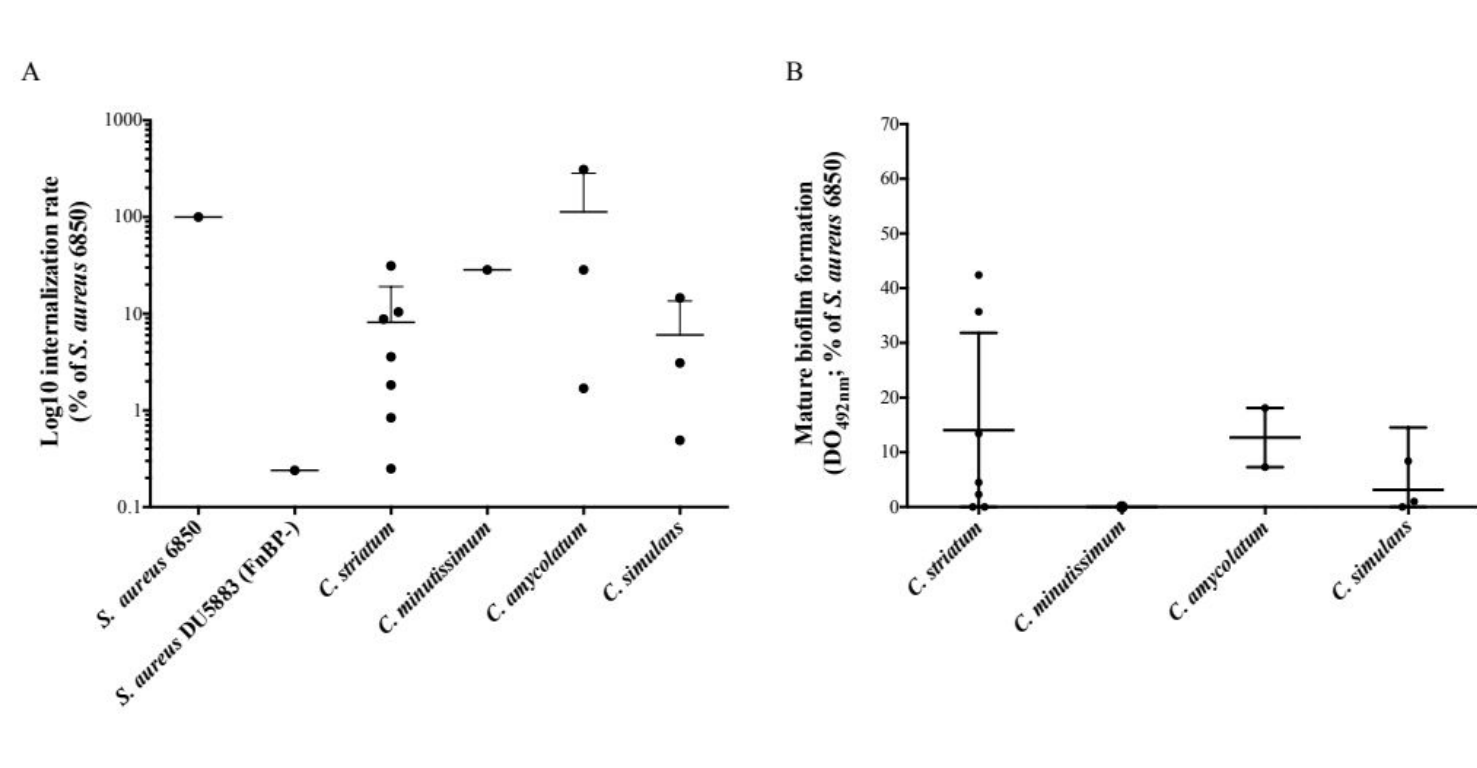

Supplement: Supplementary Figure 1 — Comparison of Corynebacterium species regarding their ability to invade MG63 human osteoblasts (A) and to form mature biofilm (B). [file Presentation_1.pptx]
