# Supplementary material for: Bone and Joint Infection Involving Corynebacterium spp.: From Clinical Features to Pathophysiological Pathways
Source: Front Med (Lausanne). 2021 Jan 21;7:539501. doi: 10.3389/fmed.2020.539501 (PMC7873945; doi:10.3389/fmed.2020.539501)
Supplement: Supplementary Table 1 — Clinical characteristics of the eleven patients with monomicrobial Corynebacterium spp. BJI. [file Data_Sheet_1.docx]

**Supplementary Table 1 –** Clinical characteristics of the eleven patients with monomicrobial *Corynebacterium* spp. BJI.

|  | **Sex; age** | ***Corynebacterium***  **species** | **Type** | **Chronology** | **Surgical management** | **Antimicrobial therapy**  **duration (weeks)** | **Main used**  **molecules** | **Outcome** | |
| --- | --- | --- | --- | --- | --- | --- | --- | --- | --- |
| 1 | M; 42.4 | *striatum* | PJI | Chronic | Non optimal | 59.4 (under ST) | BL, GP | Failure | Superinfection (*E. cloacae*) |
| 2 | M; 49.0 | *striatum* | ODI | Acute | Optimal | 13.0 | BL | Success |  |
| 3 | M; 59.1 | *simulans* | PJI | Chronic | Optimal | 22.4 | BL, CLI | Failure | Undocumented persistent infection |
| 4 | M; 51.9 | *striatum* | ODI | Chronic | Optimal | 30.1 | GP | Success |  |
| 5 | F; 65.9 | *tuberculostearicum* | PJI | Chronic | Optimal | 18.9 | GP, LZD | Success |  |
| 6 | M; 15.6 | *accolens* | PJI | Chronic | Non optimal | 110.3 (under ST) | BL, LZD, FQ | Failure | Undocumented persistent infection |
| 7 | M; 66.2 | *striatum* | ODI | Acute | Optimal | 26.3 | BL, FQ | Success |  |
| 8 | F; 87.9 | *tuberculostearicum* | PJI | Chronic | Non optimal | 135.0 (under SY) | DAP, DOX | Failure | Undocumented persistent infection |
| 9 | M; 47.5 | *simulans* | PJI | Chronic | Optimal | 18.1 | GP, CLI | Success |  |
| 10 | M; 69.7 | *urealyticum* | Native OM | Chronic | Optimal | 16,0 | BL | Failure | Undocumented persistent infection |
| 11 | M; 41.5 | *tuberculostearicum* | ODI | Chronic | Non optimal | 20.0 | GP | Failure | *Corynebacterium* persistent infection  + superinfection (*S. epidermidis*) |

*BJI, Bone and joint infection; BL, Betalactam; CLI, Clindamycin; DAP, Daptomycin; DOX, Doxycyclin; F, Female; FQ, Fluoroquinolone; GP, Glycopeptide; LZD, Linezolid; mCCI, modified Charlson comorbidity index; M, Male; ODI, Osteosynthesis device-associated infection; OM, Osteomyelitis; PJI, Prosthetic joint infection; ST, Suppressive therapy.*

**Supplementary Table 2 –** Description of patients according to the BJI types, and comparison of osteosynthesis device-related and prosthetic joint infection.

|  | | | **Native BJI** | **PJI** | **ODI** | ***p*-value** |
| --- | --- | --- | --- | --- | --- | --- |
| **n** | | | **13** | **15** | **23** |  |
| **Demographics** | | |  |  |  |  |
|  | Male gender | | 11 (84.6%) | 8 (53.3%) | 17 (73.9%) | 0.191 |
|  | Age (median, 95%CI), years | | 55.7 (45.3-64.6) | 60.9 (49.3-73.6) | 50.5 (41.5-67.5) | 0.121 |
| **Comorbidities** | | |  |  |  |  |
|  | ASA score (median, 95%CI) | | 2 (1-2) | 2 (1-3) | 1 (1-2) | 0.329 |
|  | CCI (median, 95%CI) | | 1 (1-4) | 3 (1.5-4) | 1 (0-3) | 0.078 |
| ***Corynebacterium* species** | | |  |  |  |  |
|  | *C. striatum* | | 3 (23.1%) | 6 (40.0%) | 9 (40.9%) | 0.956 |
|  | *C. tuberculostearicum* | | 0 (0%) | 3 (20.0%) | 3 (13.6%) | 0.670 |
|  | *C. simulans* | | 2 (15.4%) | 2 (13.3%) | 1 (4.5%) | 0.554 |
|  | *C. jekeium* | | 2 (15.4%) | 0 (0%) | 2 (9.1%) | 0.505 |
|  | *C. minutissimum* | | 2 (15.4%) | 0 (0%) | 2 (9.1%) | 0.505 |
|  | *C. amycolatum* | | 0 (0%) | 1 (6.7%) | 3 (13.6%) | 0.633 |
|  | *C. urealyticum* | | 2 (15.4%) | 0 (0%) | 0 (0%) | NC |
|  | Others | | 2 (15.4%) | 2 (13.3%) | 2 (9.1%) | 1.000 |
| **Plurimicrobial infection** | | | 12 (92.3%) | 9 (60.0%) | 19 (82.6%) | 0.122 |
| **BJI mechanism** | | |  |  |  |  |
|  | Superinfection | | 8 (61.5%) | 2 (46.7%) | 9 (39.1%) | 0.646 |
|  | Inoculation mechanism | |  |  |  |  |
|  |  | Postoperative | 11 (84.6%) | 14 (93.3%) | 23 (100%) | 0.395 |
|  |  | Posttraumatic | 7 (53.8%) | 1 (6.7%) | 15 (65.2%) | 0.001 |
| **BJI chronology** | | |  |  |  |  |
|  | Early infection (< 3 months) | | 9 (69.2%) | 10 (71.4%) | 15 (68.2%) | 1.000 |
|  | Chronic infection (> 4 weeks) | | 12 (92.3%) | 14 (93.3%) | 19 (82.6%) | 0.630 |
| **Diagnostic features** | | |  |  |  |  |
|  | Sinus tract | | 8 (61.5%) | 8 (72.7%) | 13 (59.1%) | 0.703 |
|  | Abscess | | 3 (23.1%) | 1 (10.0%) | 5 (22.7%) | 0.637 |
|  | Biological inflammatory syndrome | | 4 (40.0%) | 12 (92.3%) | 14 (70.0%) | 0.202 |
|  |  | Initial plasmatic CRP level (mg/L) | 56.0 (50.5-91.5) | 21.7 (10.0-79.9) | 37.3 (18.8-136.3) | 0.183 |
| **Surgical management** | | | 11 (84.6%) | 14 (93.3%) | 22 (95.7%) | 1.000 |
|  | Inappropriate surgical management | | 1 (7.7%) | 1 (6.7%) | 5 (21.7%) | 0.285 |
|  | Flap coverage requirement | | 3 (23.1%) | 0 (0%) | 5 (21.7%) | 0.136 |
| **Medical management** | | |  |  |  |  |
|  | Antimicrobial therapy duration | |  |  |  |  |
|  |  | Total treatment duration (weeks) | 21.1 (13.9-66.7) | 23.1 (18.1-53.9) | 25.6 (13.1-41.1) | 0.604 |
|  |  | *Corynebacterium*-specific treatment duration | 16.0 (12.7-20.0) | 20.3 (15.7-40.3) | 14.9 (13.1-24.6) | 0.138 |
|  | *Corynebacterium*-specific IV treatment | | 13 (100%) | 15 (100%) | 20 (87.0%) | 0.264 |
|  |  | IV treatment duration | 14.5 (4.2-16.1) | 13.7 (11.4-15.3) | 14.3 (12.6-22.0) | 0.464 |
|  |  | Oral switch | 9 (69.2%) | 6 (40.0%) | 11 (55.0%) | 0.500 |
|  | *Corynebacterium*-specific combination therapy | | 10 (83.3%) | 10 (66.7%) | 16 (76.2%) | 0.509 |
|  |  | Combination therapy duration | 13.9 (6.1-17.9) | 12.9 (9.8-26.4) | 12.6 (7.6-14.4) | 0.750 |

*ASA, American society of anesthesiologists; BJI, Bone and joint infection ; CCI, Charlson comorbidity index; CRP, C-reactive protein; IV, Intravenous ; ODI, Osteosynthesis device-related infection; PJI, Prosthetic joint infection.*
